# Sharpening the focus: differentiating between focus groups for patient engagement vs. qualitative research

**DOI:** 10.1186/s40900-018-0102-6

**Published:** 2018-06-25

**Authors:** Nicole Doria, Brian Condran, Leah Boulos, Donna G. Curtis Maillet, Laura Dowling, Adrian Levy

**Affiliations:** 1 Maritime SPOR SUPPORT Unit, 5790 University Avenue, Halifax, NS B3H 1V7 Canada; 2 Department of Community Health and Epidemiology, Dalhousie University, 5790 University Avenue, Halifax, NS B3H 1V7 Canada

**Keywords:** Patient-oriented research, Strategy for patient-oriented research, Qualitative research, Patient engagement, Focus groups, Discussion groups

## Abstract

**Plain English summary:**

Patient engagement is an opportunity for people with experience of a health-related issue to contribute to research on that issue. The Canadian Strategy for Patient-Oriented Research (SPOR) highlights patient engagement as an important part of health research. Patient engagement, however, is a new concept for many researchers and research ethics boards, and it can be difficult to understand the differences between patient engagement activities and research activities. *Focus groups* are one example of how research and patient engagement activities are often confused.

We distinguish these two types of activities by using different terms for each. We use *focus groups* to refer to research activities, and *discussion groups* to refer to patient engagement activities. In focus groups, researchers collect data by speaking with a group of research subjects about their experiences. Researchers use this information to answer research questions and share their findings in academic journals and gatherings. In patient engagement, discussion groups are a way for patients to help plan research projects. Their contributions are not treated as research data, but instead they help make decisions that shape the research process. We have found that using different language to refer to each type of activity has led to improved clarity in research planning and research ethics submissions.

**Abstract:**

**Background**

In patient-oriented research (POR), focus groups can be used as a method in both qualitative research and in patient engagement. Canadian health systems researchers and research ethics boards (REBs), however, are often unaware of the key differences to consider when using focus groups for these two distinct purposes. Furthermore, no one has clearly established how using focus groups for these two purposes should be differentiated in the context of Canada’s Strategy for Patient-Oriented Research (SPOR), which emphasizes appropriate patient engagement as a fundamental component of POR.

**Body**

Researchers and staff in the Maritime SPOR SUPPORT Unit refer to focus groups in patient engagement as *discussion groups* for clarity, and have developed internal guidelines to encourage their appropriate use. In this paper, the guidelines comparing and contrasting the design and conduct of focus groups and of discussion groups is described, including: the theoretical framework for each; the need for research ethics board review approval; identifying participants; collecting and analyzing data; ensuring rigour; and disseminating results.

**Conclusion**

The MSSU guidelines address an important and current methodological challenge in patient-oriented research, which will benefit Canadian and international health systems researchers, patients, and institutional REBs.

## Background

### Patient engagement in the context of Canada’s strategy for patient-oriented research

Patient engagement is a new area of activity for many Canadian health researchers and institutional research ethics boards (REBs). Launched in 2011, the Strategy for Patient-Oriented Research (SPOR) is a pan-Canadian initiative intended to “foster evidence-informed health care by bringing innovative diagnostic and therapeutic approaches to the point of care, so as to ensure greater quality, accountability, and accessibility of care” [1]. The core concepts of SPOR are described in Table 1. SPOR highlights patient engagement as a fundamental element of patient-oriented research (POR) [1]. In this context, patient engagement is not a research method, but rather a process for involving patients as partners in the planning and conduct of research.

**Table 1:** Glossary

**Patient-oriented research (POR)** Health research that “engages patients as partners, focusses on patient-identified priorities and improves patient outcomes” [2] with the goal of advancing healthcare systems and practices [2].	
**Strategy for Patient-Oriented Research (SPOR)** A national program launched in 2011 by the Canadian Institutes of Health Research (CIHR) and provincial/territorial partners to support patient-oriented research (POR) in Canada.	
**Support for People and Patient-Oriented Research and Trials (SUPPORT) Units** Local clusters of specialized research and knowledge resources that provide the necessary expertise to pursue patient-oriented research (POR) successfully [1].	
**Patient engagement** Active collaboration with patients across all phases of research, including planning, conducting, and disseminating results [2]. A core element of patient engagement is that persons with experience of a health-related issue (e.g. illness, interaction with health care system) contribute in a meaningful way to research on that issue.	
**Patient** “An overarching term inclusive of individuals with personal experience of a health issue and informal caregivers, including family and friends” [2]	

While partnerships between patients, communities, researchers and other stakeholders are not new in Canada [2], SPOR promotes these partnerships in areas of research where they are not as common. For example, SPOR funding competitions require patients be included as members of research teams for an application to be considered eligible. For some SPOR-funded researchers, these studies represent their first experience working with patients as partners in research. To support uptake of the collaborative principles of POR, SUPPORT Units are mandated to advance the science of patient engagement by supporting meaningful involvement of patients across all phases of research [3]. Commitment to this endeavour provides the foundation for including patient engagement in health research that is aimed at improving health outcomes for Canadians.

### The distinction between activities in research and patient engagement is not always clear

Distinguishing between research and patient engagement can be challenging for health researchers and REBs. Part of the confusion stems from the fact that similar activities can be used in both research and patient engagement, and such confusion impedes advancements in the area of POR. Further, when engagement is implemented inappropriately, patients can be exposed to undue risk; harmonious relationships between researchers and patients can be jeopardized; and researchers can be discouraged from adopting a patient-oriented approach.

Focus groups are a common example of how the overlap between research and patient engagement methods can be confusing. In qualitative research, focus groups are used as an interview method for data collection [4]. However, while researchers may directly interact with patients to collect focus group data, reporting on this interaction as evidence of patient engagement is erroneous [5]. In patient engagement, focus groups are not used to collect research data, but instead are used as a method to direct and inform research decisions by consulting with patients and applying their expertise [5, 6].

In addition to these differences, the design of both uses of focus groups requires a variety of distinct considerations, including: identifying the purpose; recruiting participants; collecting and using data; and REB approval [5, 7]. The lack of published guidelines clearly laying out these differences can adversely affect researchers, patients, and REBs [5]. Canadian REBs are increasingly facing issues in relation to patient engagement in POR, which is resulting in inconsistent interpretation and application of REB review requirements. Just as researchers may not know when their focus group plans require REB approval, REBs may similarly be unsure.

## Main text

### One remedy is to establish clear terminology and design

To differentiate between focus groups in qualitative research and patient engagement, researchers and staff in the Maritime SPOR SUPPORT Unit (MSSU) refer to focus groups in patient engagement as *discussion groups*. Through the MSSU’s auspices, informal guidelines comparing focus groups and discussion groups have been developed to encourage appropriate use. This paper expands on the MSSU guidelines to serve as an example for other organisations, REBs, and health systems researchers. This example is intended to help those new to patient engagement and/or qualitative research as they plan their activities. It is also intended to encourage discussion among those more familiar with qualitative research and patient engagement, particularly in regards to how we think about interactions between patients and researchers in different research contexts.

### Comparing focus groups and discussion groups

**Fig. 1:**
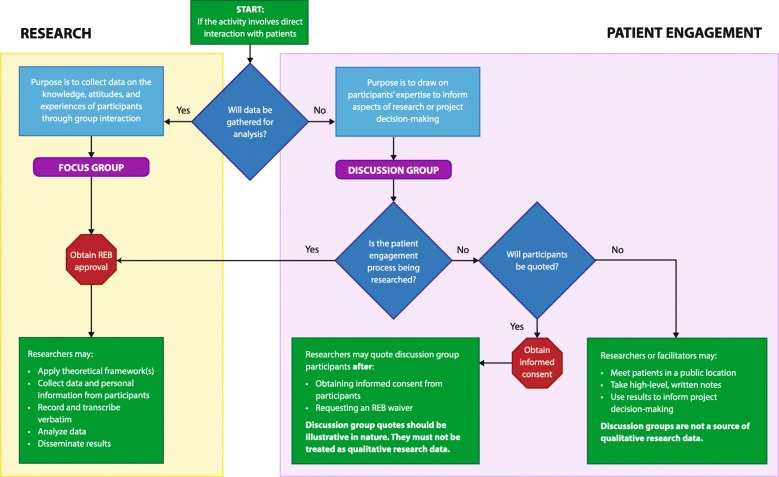
Research patient engagement

The purpose of using focus groups in qualitative research is to collect data on the knowledge, attitudes, and experiences of participants through group interaction [4, 7–10]. In patient engagement, discussion groups are used as a type of focus group to draw on the knowledge of patients to inform various aspects of research or project decision-making. The term discussion group indicates that design criteria have been selected to ensure patients are involved as contributors to research decision-making [11]. This contrasts with the objective of traditional focus groups, in which participants are involved as research subjects who provide data that answers research questions [12]. Given that SPOR encourages engagement of patients as “experts from their unique experience” [3], discussion groups emphasize co-learning, multi-way communication, and collaboration. Fig 1 shows a series of steps in the form of a decision model to guide researchers on whether a focus group or discussion group is more appropriate. Table 2 presents a summary of the core characteristics differentiating focus groups and discussion groups.

**Table 2:** Comparison of Maritime SPOR SUPPORT Unit design criteria for focus and discussion groups^a^

Level of Distinction	Characteristic	Focus Groups	Discussion Groups
High	Type of participant	Research participant - An individual whose data, or responses to interventions, stimuli, or questions by a researcher are relevant to answering a research question; also referred to as “human participant,” and in other policies/guidance as “subject” or “research subject”	Patient Contributor – An individual who possesses relevant personal experience of the health condition in question, and/or as a caregiver. Patient contributors draw on this experience to inform research decision making
High	Purpose	Qualitative research method for data collection on the knowledge, attitudes, and experiences of participants through group interaction	Tool for patient engagement to draw on patients’ expertise to inform aspects of research or project decision-making
Medium	Prior consultation and/or approval	Required – Project principal investigator; appropriate institutional research ethics board (REB)	Required – Project principal investigator; patient engagement/facilitation expert
Low	Monitored	Required – Project principal investigator or approved research team member	Required – Project principal investigator or approved research team member; patient engagement expert
Low	Identification of participants	Participants are formally recruited and invited to participate based on their shared experiences as related to research objectives	Patients are invited to participate based on their lived experience and expertise relevant to the project objectives
Low	Location	Location must be approved by REB. Public locations such as community centers or libraries are ideal; private and comfortable locations within academic or institutional settings are alternative options. Video or teleconferencing may also be used	Public locations such as community centers or libraries are ideal; private and comfortable locations within academic or institutional settings are alternative options. Video or teleconferencing may also be used
High	How information and/or data is collected	With REB approval and informed consent from participants, data collection can be audiotaped, transcribed verbatim, videotaped, or recorded using field notes. Data is collected by a non-participating moderator (e.g. researcher, qualified staff, research assistant, graduate student, etc.) using an interview guide to facilitate discussion	Collaborative and interactive forms of recording are ideal. These can include written transcripts/notes, shared note-taking (e.g. flip-charts), participant notes, and comments through activities (e.g. card storming)
Low	Compensation	Financial compensation (money or a gift card) is the most common type of incentive for participation. Research participants are compensated at the conclusion of the focus group and each participant receives equivalent payment. Providing a financial incentive offers several benefits, such as: compensating individuals for travel and time, recognizing the value of the participants, decreasing recruitment time, and increasing accountability of participants	MSSU reimburses travel expenses (e.g. mileage, parking) for discussion group contributors. Nationally, SUPPORT Units and Networks are increasingly compensating patients for participation in engagement activities. The amount and form of compensation depends on the nature of the activity in question; for discussion groups, amounts can range from $30 to $50
High	How information and/or data is used and/or analyzed	Transcripts, written notes and audio/video recordings (transcribed verbatim) are analyzed using qualitative research techniques. Data analysis may include reading and coding of transcripts, information classification, and identification of quotes and/or examples of themes for research results	Information is used to support research or project decision-making (e.g. a list of patient-made recommendations for researchers), or patients make decisions directly. No data analysis takes place
High	Research ethics board (REB) review	Required – As outlined by the Tri-Council Policy Statement, approval must be obtained prior to any focus group work (including recruitment, planning sessions, and/or pilot)	Should not be required – The Tri-Council Policy Statement does not cover patient engagement. Patients are involved as subject matter experts to consult on a project and are not research subjects
Medium	Written statement of intent	Required – Informed consent requires a plain language description of the research project with any further documentation as required under REB approval, which must be delivered to participant(s) prior to participation	Required – A plain language description of the activities and their relation to the research project must be provided prior to participation
Low	Confidentiality agreement	Required – Describe procedures for maintaining confidentiality in informed consent process	Required – Discuss confidentiality with participant(s) and obtain written or verbal acknowledgment of agreement
Low	Right to withdraw	Yes, at any time; details subject to REB approval	Yes, at any time
Medium	Gathering of personal information (PI)	Permitted – PI may be collected during focus groups as approved by REB and provincial privacy legislation	Permitted, for administrative purposes only – PI such as contact information may be collected for administrative purposes with consent
High	Dissemination	May include written reports, scholarly journal articles, conference presentations, and/or other forms of dissemination	A summary report should be shared with participants, indicating what feedback was taken, what was not, and why

^a^Adapted from Table 8.1 in Krueger, R.A. & Casey, M.A., Focus Groups: A Practical Guide for Applied Research, Los Angeles: Sage, 2015, p. 182–184

#### Design

Focus groups are used in health services research in both single method and multi method designs [7, 8, 10]. The optimal size for a focus group is between four and 12 participants, which creates a large enough group to facilitate discussion without inhibiting balanced participation [7, 9]. Participants are recruited and invited to participate based on their shared experiences related to the research questions and objectives [4, 7]. Having a homogenous group facilitates a narrative of shared experiences, fosters group comfort and cohesion, and improves the quality of group interaction [4, 7, 9, 13].

In comparison, discussion groups are used in health services research as a patient engagement activity. Shippee et al. [14] describe how patients can contribute in the planning, execution and/or translational phase of a research study. Patients who take part in discussion groups (patient contributors) are identified based on relevant personal experience of the health condition in question, and/or on their experience as caregivers. As with focus groups, patient contributors are selected based on having some common experience or shared social or geographic community [15]. Recruitment is often conducted through a combination of approaches similarly used in qualitative research, including: posters, social media promotion, referrals through clinical settings, and dissemination of recruitment messages through community organizations and leaders [16].

#### Collection of data and/or information

In focus groups, data are collected by a moderator who is often the researcher or part of the research team [4, 7, 13]. Typically done using an interview guide with open-ended questions, the moderator guides and directs the conversation, elicits discussion, and encourages the interest and comfort of participants [4, 8]. The moderator guides the group without actively participating or expressing their own views [8, 9], and having a skilled moderator is critical to a successful focus group [7, 17]. Focus groups are generally held in public locations that are comfortable, relaxed, and non-threatening [4, 7, 10]. With the consent of participants, focus groups can be audiotaped and transcribed verbatim to facilitate later data transcription, compilation and analysis [4, 7, 8, 10, 18]. Focus groups are also sometimes videotaped to observe the interaction of participants [7, 8]. For focus group participation, financial compensation (money or a gift card) is the most common type of incentive [7]. Generally, research participants are compensated at the conclusion of the focus group and each participant receives equivalent payment [7]. Providing a financial incentive offers several benefits, such as compensating individuals for travel and time, recognizing the value of the participants, decreasing recruitment time, and increasing accountability of participants [7].

Discussion groups should likewise be conducted by trained and experienced facilitators who have led similar activities such as workshops for non-research purposes [15]. The facilitator should clearly articulate their own role, the role of contributors, and how the outcomes from the discussion group will inform research-based decisions [5, 14]. A typical discussion guide includes an overview of the research project and describes the objectives of the engagement initiative. Led by the facilitator, contributors develop a list of recommendations that address the objectives of the engagement initiative. Throughout this process, the facilitator and contributors engage in co-learning to develop a shared understanding of the research project, determine the research needs from a patient perspective, and make protocol recommendations [14]. Given discussion groups gather input on decisions rather than explore contributors’ experiences in depth, detailed record keeping is not required. Rather than relying on audio or visual recording, interactive and participatory methods (e.g. shared note-taking or card-storming) are more appropriate methods to record discussion highlights. MSSU reimburses travel expenses (e.g. mileage, parking) for discussion group contributors. Nationally, SUPPORT Units and Networks are increasingly compensating patients for participation in engagement activities. The amount and form of compensation depends on the nature of the activity in question; for activities similar to discussion groups, amounts can range from $30 to $50 [19].

#### Analysis of data and/or information

The analysis of focus group data is a purposeful and sequential process that should be systematic and verifiable [7]. Qualitative research techniques and frameworks are typically used to direct the analysis and interpretation of the focus group data [4]. The purpose of the research will guide the choice of qualitative framework used, as well as the direction, depth and intensity of analysis [7]. With focus group data, analysis must consider the impact of the group dynamic/interaction and not just individual responses [8, 10]. Analysis generally involves transcribing the discussion, developing a coding framework for the transcripts, classifying information into themes, and gathering quotes and/or examples from the text to support these themes [4, 7, 20]. To validate the findings, two or more researchers often independently analyze data and then compare and validate the codes and themes they identified [7]. Computer software may be used for data analysis [7, 20].

Discussion groups do not produce research data that are thematically analyzed and, therefore, research techniques and frameworks are not necessary. Rather than analysis, real-time processing takes place during the interaction and co-learning with patient contributors. The output of these activities is generally presented as a list of recommended options that the research team draws upon when making decisions about the study. There is no need for the use of analytical software.

#### Rigour

In research, strategies can be applied during planning and implementation to ensure the rigour of focus group findings. Strategies include running a pilot-test of interview questions; securing a trained moderator for facilitation; recording data accurately; having data transcribed verbatim; member-checking findings with participants; peer debriefing/reviewing of the data among research team members; keeping a detailed audit trail; validating analysis by a third party; and triangulating data [7, 9, 20, 21]. Incorporating such strategies enhance the trustworthiness and credibility of the data.

Like focus groups, discussion groups must be rigourously planned and facilitated to generate valid and useful recommendations. Given the underlying intent of patient engagement is to support decision-making, there should be a clear connection between a project’s objectives, the decisions patients are contributing to, the discussion guide, and the application of patient input [22, 23]. The benefit of engaging patients should also be clearly identifiable both to the research team and to the patient contributors [23]. Patient-researcher partnerships are a defining element of SPOR and, therefore, discussion groups should not be the only means of patient engagement in a program of research [3, 12]. In addition to informing research decision-making, discussion groups can be used as an opportunity to build relationships with patient communities and involve individual patients on future research teams. SPOR has indicators of success for patient engagement that provide useful metrics for patient engagement overall, as well as for individual engagement initiatives or activities such as discussion groups [3].

#### Results and dissemination

Results from focus groups are generally reported as qualitative research findings. Research findings are organized into main themes and supported by verbatim quotations that reflect the group conversation [20]. Results tend to be formally reported in academic literature and/or disseminated in academic forums such as research conferences [7].

In comparison, the results or outcomes of discussion groups are generally presented as an unpublished summary report that indicates what feedback was taken, what feedback was not, and why [11, 14, 15]. This report is shared with all patients who contributed to the discussion group, and emphasizes the link between recommendations offered by patients and the research decisions that were made. This is a critical step of patient engagement as it develops lines of communication between the research team and patient population/community in question [11, 14, 15]. In contrast to focus group data, the publication of discussion group outcomes is secondary to their use in research decision-making. There is, however, a need to develop a body of peer-reviewed literature on patient engagement [14]. When preparing manuscripts for publication, authors should describe how discussion groups contributed to a study’s development [22]. Guidelines have been developed to support the reporting of patient involvement in research [22, 23], but at this stage in their development these standards “are conceptualized within the culture and language of research” [23]. As a result, they do not always draw clear distinction between description of research and patient engagement activities. Future iterations of these guidelines should offer explicit guidance on differentiating between patient engagement and research.

#### REB approval

The Tri-Council Policy Statement (TCPS2) requires qualitative research protocols involving human subjects to undergo review and approval by an REB. The TCPS2 does not, however, directly address patient engagement [24]. Given the purpose of patient engagement is *not* to “extend knowledge” [24], discussion groups should not require REB review when properly planned and conducted. This issue is currently interpreted differently between institutional REBs, with some requiring engagement initiatives to undergo review and others waiving this requirement. This situation parallels the distinction between quality improvement and research [24], and similar clarity is needed to address the inconsistent interpretation and application of REB requirements for patient engagement.

Whether REB review is required or not, there are ethical considerations that must be addressed during planning and implementation of discussion groups. A full discussion of these considerations is beyond the scope of this paper (see Shimmin et al. [25] and Pandya-Wood et al. [5] for further discussion). Two methodological considerations are offered here. First, inclusion of patient engagement as an element of research planning, or to direct research conduct, should still be described in the REB application for the project they support [26]. Second, teams who wish to publish outcomes of discussion groups in peer-reviewed journals should demonstrate that REB review took place (e.g. for research supported by discussion groups) or that review was not required.

## Conclusion

In Canada, and internationally, it has been established that research and patient engagement are separate activities, differing in whether patients are involved as research subjects or as contributors to the research process [3, 4]. Confusion arises when there is methodological overlap between these fields, as is the case with focus groups. The MSSU has found that using the terms *focus group* and *discussion group* to differentiate between research and patient engagement methods has led to improved clarity in research planning. This paper highlights important methodological and practical considerations to support this distinction, and establishes discussion groups as a distinct activity for patient engagement.

This paper does not address several related issues, including the overlap between research and patient engagement in other methods of qualitative data collection (e.g. one-on-one interviews, surveys) or in other common approaches (e.g. participatory action research, program evaluation). This paper also does not provide guidance on how to conduct research on patient engagement or how to evaluate the impact of patient engagement on research projects. Although beyond the scope of this paper, these areas of study show promise for future research. Finally, the authors acknowledge that the term ‘patient’ is contested, and that individual research teams will differ in how they conceptualize the inclusion of lived experience as a form of expertise. The MSSU uses the term patient to reflect the preferred language of SPOR. Fully exploring perspectives about the appropriateness of this term is outside the scope of this paper.

With regards to the inconsistent application of REB requirements, there is a need for these requirements to be addressed regionally and nationally as SPOR matures. Initiatives are underway to address this issue (e.g. Dubois-Flynn [27]; NL SUPPORT, & Health Research Ethics Authority [28]), and it should be considered whether REBs are the appropriate body to review patient engagement. For example, SPOR mandates that patients be engaged in research during priority-setting and planning; this stage of research traditionally occurs prior to REB review. If REB review is deemed necessary for patient engagement, then changes to traditional REB processes need to be made. True to the values of SPOR, patients, researchers, and REBs should collaborate to navigate this change.

## Abbreviations

MSSU: Maritime SPOR SUPPORT Unit
POR: Patient-oriented research
REBs: Research ethic boards
SPOR: Strategy for Patient-Oriented Research
SUPPORT: Support for People and Patient-Oriented Research and Trials
